# Systematic comparison of transcriptomes of Caco-2 cells cultured under different cellular and physiological conditions

**DOI:** 10.1007/s00204-022-03430-y

**Published:** 2023-01-21

**Authors:** Janneke Elzinga, Menno Grouls, Guido J. E. J. Hooiveld, Meike van der Zande, Hauke Smidt, Hans Bouwmeester

**Affiliations:** 1 Laboratory of Microbiology, Wageningen University and Research, Wageningen, The Netherlands; 2 Division of Toxicology, Wageningen University and Research, Wageningen, The Netherlands; 3 Nutrition, Metabolism and Genomics Group, Division of Human Nutrition and Health, Wageningen University and Research, Wageningen, The Netherlands; 4 Wageningen Food Safety Research, Wageningen University and Research, Wageningen, The Netherlands

**Keywords:** Transcriptome, Caco-2, In vitro, 3R, Gut-on-a-chip, Good in vitro method practices

## Abstract

There is a need for standardized in vitro models emulating the functionalities of the human intestinal tract to study human intestinal health without the use of laboratory animals. The Caco-2 cell line is a well-accepted and highly characterized intestinal barrier model, which has been intensively used to study intestinal (drug) transport, host–microbe interactions and chemical or drug toxicity. This cell line has been cultured in different in vitro models, ranging from simple static to complex dynamic microfluidic models. We aimed to investigate the effect of these different in vitro experimental variables on gene expression. To this end, we systematically collected and extracted data from studies in which transcriptome analyses were performed on Caco-2 cells grown on permeable membranes. A collection of 13 studies comprising 100 samples revealed a weak association of experimental variables with overall as well as individual gene expression. This can be explained by the large heterogeneity in cell culture practice, or the lack of adequate reporting thereof, as suggested by our systematic analysis of experimental parameters not included in the main analysis. Given the rapidly increasing use of in vitro cell culture models, including more advanced (micro) fluidic models, our analysis reinforces the need for improved, standardized reporting protocols. Additionally, our systematic analysis serves as a template for future comparative studies on in vitro transcriptome and other experimental data.

**Supplementary Information:**

The online version contains supplementary material available at 10.1007/s00204-022-03430-y.

## Introduction

After (partial) digestion of food and absorption of fluid, nutrients and drugs in the upper part of the human gastrointestinal tract (GIT), the chyme reaches the colon, where fluid and electrolytes are (re)absorbed, whereas the small intestine is the most important one for the uptake of food-related chemicals and nutrients. The colon has other essential functions related to human health (Silverthorn et al. 2016). The colon abundantly contains microorganisms, estimated to reach a total of ~ 10^12^ microorganisms (Sender et al. 2016), which aid in the transformation of food components, *e.g.,* yet undigested complex carbohydrates, to compounds, such as short-chain fatty acids and vitamins, which contribute to host health (Salvador et al. 1993). Moreover, intestinal microorganisms have demonstrated a significant impact on drug transformation (Sousa et al. 2008). The role of the chemical exposure and human intestinal microbiota in various diseases, ranging from intestine-related diseases, including Inflammatory Bowel Disease (Derrien et al. 2017; Lomer et al. 2002; Parada Venegas et al. 2019), neuronal diseases, such as Parkinson’s and Alzheimer’s (Chen et al. 2022; Sun et al. 2018), and cancer (Abreu and Peek 2014; Arthur et al. 2012; Dihal et al. 2007), to metabolic and psychological disorders (Allen et al. 2017; Hartstra et al. 2014; Sarkar et al. 2018; Singer-Englar et al. 2019), has sparked the interest in human intestinal health. In this context, there is an increasing need for in vitro and in vivo models reliably mimicking the human GIT, to investigate intestinal barrier integrity, host–microbe interactions and the toxicological effects of food-related chemicals, food components, bacteria-derived metabolites, and drugs.

Murine and porcine in vivo models have been commonly used (Etienne-Mesmin et al. 2019; Gustafsson et al. 2012; Yissachar et al. 2017) to answer a wide range of scientific questions related to human intestinal health. Although those models allow for experiments in the context of the whole organism, they lack translational value in terms of human (intestinal) physiology (Mak et al. 2014; McGonigle and Ruggeri 2014), anatomy (Kararli 1995; Thompson and Trexler 1971), and microbiology (Faith et al. 2010; Nguyen et al. 2015; Turnbaugh et al. 2007). This further strengthens the existing ethical concerns about the use of animals for safety and efficacy testing of compounds of human interest (Ferdowsian and Beck 2011). More than ever, there is a call for increased insight in existing in vitro models mimicking the human intestinal tract as well as for improved in vitro models. This may not only help to refine protocols of dedicated animal studies, but also *reduce* the number of animals sacrificed for science. Eventually and more importantly, the use of in vitro models might partially replace the use of animal models (Rahman et al. 2021; Russell 1959).

The immortalized cell line Caco-2 is a well-accepted and highly characterized model for the human intestinal epithelium. This cell line was originally isolated in the 1970s from a colorectal tumor (Fogh et al. 1977). As opposed to other isolated colon carcinoma cell lines (Chantret et al. 1988), Caco-2 cells demonstrated spontaneous differentiation upon long-term culture leading to expression of several morphological and biochemical characteristics of small intestinal enterocytes (Matsumoto et al. 1990; Pinto et al. 1983). Growth and differentiation of Caco-2 cells on permeable membranes allow investigation of the transport properties of the cells (Wilson et al. 1990), and this model has been extensively applied and reported in transport studies for toxicological or pharmaceutical research (Bouwmeester et al. 2011; Brand et al. 2008; Hidalgo et al. 1989; Hubatsch et al. 2007; Wilson et al. 1990; Yamashita et al. 2000). Additionally, from the parental Caco-2 cell line, several clones have been generated over the years and selected based on characteristics of interest (reviewed in Sambuy et al. 2005) (Sambuy et al. 2005). Besides Caco-2 cells, other human intestinal cell lines have also been commonly used as a model of the human intestinal epithelium, including T84 (Donato et al. 2011) and HT29(-MTX) cells (Elzinga et al. 2021; Hilgendorf et al. 2000; Lefebvre et al. 2015; Lesuffleur et al. 1990; Zweibaum et al. 2011).

Despite the extensive use of the Caco-2 cell line in commonly used simple cell culture inserts (*e.g.,* Transwell, ThinCert), its representativeness of the human intestinal epithelium has been debated (Delie and Rubas 1997; Press and Di Grandi 2008). In this respect, advanced in vitro techniques including the use of microfluidic devices (Kim and Ingber 2013; Shah et al. 2016) and co-culture with other human cell types or (anaerobic) bacteria (Jalili-Firoozinezhad et al. 2019; Kampfer et al. 2017; Kim et al. 2016) have been applied to this cell line to mimic the intestinal tract more accurately in terms of physiology, cell differentiation, drug transport and/or host–microbe interactions (Jalili-Firoozinezhad et al. 2019; Kampfer et al. 2017; Kim and Ingber 2013; Kim et al. 2016; Shah et al. 2016). Simultaneously, primary epithelial cell cultures, including enteroids and adult and induced pluripotent stem cell-derived intestinal models have been developed (Huch et al. 2017; Janssen et al. 2020; McCracken et al. 2011; Sato et al. 2011, 2009; Spence et al. 2010) and may be used as alternative to more complex in vitro intestinal models depending on the research question. Although these more complex models allow the development of personalized models of the human intestinal tract, the power of Caco-2 cells grown on permeable membranes lies in their culture simplicity, reproducibility, and the considerable number of studies available for comparison. Consequently, in theory, its widespread use should allow systematic comparison of the effects of different culturing parameters on the intestinal cellular response. Such a comparison would not only help to assess the reproducibility of in vitro models using Caco-2 cells, but also provide suggestions for adjustments of current in vitro techniques to improve their functionality and to better conform to the OECD Guidance Document on Good In Vitro Method Practices (GIVIMP) (OECD 2018).

In this study, we aimed to compare cellular responses of different Caco-2 cell-based in vitro models based on gene expression, in which in vitro “model” is specified as “the physical and cellular conditions under which the cells are cultured”. We focused only on studies in which transcriptome analysis was performed on Caco-2 grown on permeable membranes, since this outcome allows for a comprehensive description of the cell response and serves as a starting point for investigating other outcomes. We collected published studies on Caco-2 cells cultured as cell layers in cell culture inserts or in devices, such as microfluidic chips, as well as studies with more biologically complex models in which Caco-2 cells were cultured as spheres, co-cultured with other cell types in a different compartment or exposed to human intestinal bacteria or their products. Based on the collected studies (2007–2021), we defined eight relevant experimental variables and utilized a bioinformatics approach to analyze and interpret the effect of these variables on transcriptomic responses. We followed an unbiased approach to explore the contribution of the defined variables to the overall transcriptome profiles. Subsequently, we zoomed in on specific genes and corresponding pathways and biological processes, of which regulation of expression could to a significant extent be explained by one of the variables. Additionally, to complement transcriptome data, we carefully extracted other experimental parameters and evaluated several functional experimental outcomes. Of these, only Trans-Epithelial Electrical Resistance (TEER) turned out to be commonly reported and thus was compared between respective studies. Overall, our study comprises a systematic and critical data analysis of in vitro models using Caco-2 cells grown on permeable membranes.

## Materials and methods

### Study collection

**Fig. 1:**
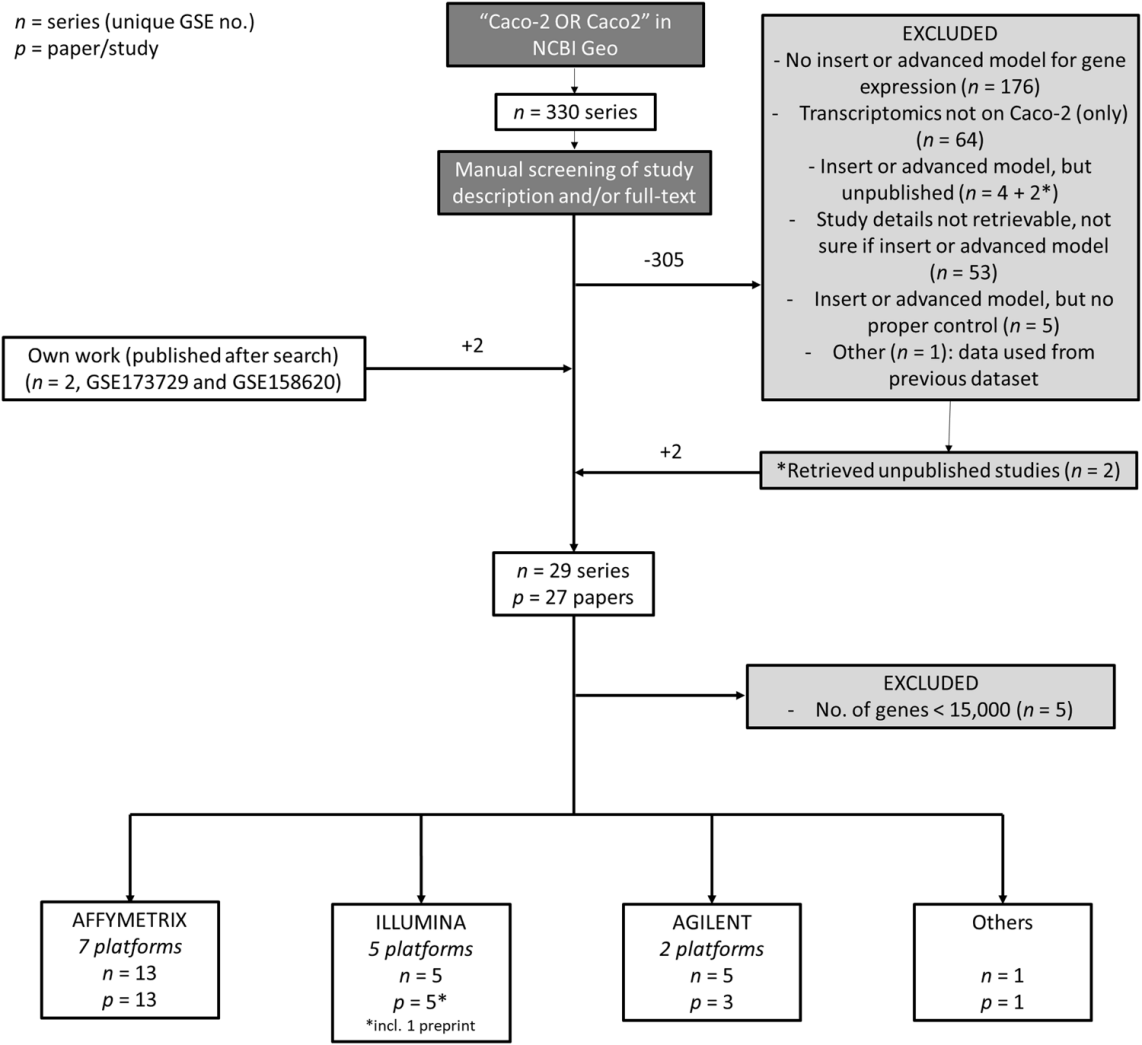
Flow chart of study selection process. The NCBI Geo Database was searched for "Caco-2" or "Caco2" in May 2021, which retrieved 330 data series. Exclusion of 306 data series and inclusion of two (own) data series resulted in a final selection of 29 series linked to 27 unique research papers. After exclusion of five data series because of limited numbers of analyzed genes, studies were divided per manufacturer, of which the Affymetrix platform comprised the largest group with 13 data series corresponding to 13 research papers

A schematic overview of the study selection and the corresponding number of series can be found in Fig. 1. The NCBI Gene Expression Omnibus (GEO) was searched for the term “Caco-2 OR Caco2” in May 2021, which resulted in 330 series with unique GEO Series identifier (GSEid) (Online Resource 1). Title, accession display and/or full-text paper of each series were manually screened to select for data series in which transcriptomic analysis was performed on Caco-2 cells which were cultured in cell culture inserts and in more advanced in vitro models with an apical and a basolateral compartment, including adapted inserts that introduce alterations, such as flow or an anoxic compartment, gut-on-chips, and 3D spheric cell models. If in doubt, the full text of publication(s) linked to the series was screened. Series were excluded for which (as primary reason) a) no cell culture insert or advanced in vitro model was used; b) transcriptomics was performed on or including other cell types than Caco-2 cells (*i.e.,* the mRNA would not only be derived from Caco-2 cells) c) no proper control condition was included (*i.e.,* not commonly used medium); d) data had been taken from a previously deposited GEO DataSet and/or; e) study details could not be retrieved (*e.g.,* studies were not published). For series of which the description in the database pointed toward the use of a cell culture insert or advanced in vitro model, but which were not linked to a publication in GEO, potential corresponding publications were actively searched using PubMed, Scopus, and Google. Additional databases (SRA-database from NCBI, as well as Array Express from EMBL-EBI) were searched using the same strategy but did not retrieve additional studies that were not already present in GEO. Lastly, we included data series from our own work, which had been submitted to NCBI but were only released after the date of the database search (GSE158620 and GSE173729). Next, array platforms on which < 15,000 unique genes were analyzed, were excluded from further analysis (Online Resource 1). Series were divided per platform manufacturer, distinguishing between Affymetrix, Illumina, Agilent, and “others”. Because of the low representation of models in the latter three categories (thus resulting in a low statistical power), only studies performed on Affymetrix platforms were included for further analysis.

### Data extraction

From all selected data series, only data of samples encompassing Caco-2 cells grown under proper control conditions (*i.e.,* regular cell medium) or Caco-2 cells exposed to non-pathogenic intestinal bacteria, or their bacterial products were selected and downloaded. Additional data were manually extracted from full-text papers. Information on experimental set-up was extracted primarily from method-sections or from the Supplementary Information. Some papers referred to previous publications for the used experimental procedures, which then were assessed as well. In case the experimental parameter of interest could not be retrieved, it was considered not reported (“NR”), except for culture temperature and atmosphere (assuming this was 37 °C at 5% CO_2_). Additionally, the seeding area of cell culture inserts was based on standard sizes, in case number of wells or other information was provided. Coating of membranes was considered not applicable (“NA”) if not reported, because this was not included in standardized Caco-2 insert protocols (Hubatsch et al. 2007; Natoli et al. 2012). Details for each study can be found in Online Resource 1. Data on TEER were manually extracted from the text and/or extracted from figures using a digital, on-screen ruler (Measura X, Gekar Tech). Values that had been normalized to a control were excluded.

Each model identified was further categorized based on “GSEid” (GEO Series identifier), “Microbiome” (exposure to non-pathogenic bacteria or their bacterial compounds), “Culture Time” (in which short (< 9 days), medium (≥ 9, but ≤ 17 days) or long (> 17 days) were distinguished), “Oxygen” status in apical compartment (anoxic or oxic), “Flow” (static, dynamic or partially dynamic), “Cell System” (Caco-2 only or co-culture), “Device” (insert or chip) and “Platform” (type of array platform used). Partially dynamic refers to models where flow was applied for the majority of the entire culture time (i.e. > 80%) and/or only either to the apical or basolateral side of the cells. In our dataset, co-cultures consisted of Caco-2 cells cultured in same device with human leukemia monocytic cell line (THP-1), peripheral blood mononuclear or endothelial cells, of which mRNA was extracted from the Caco-2 cells separately.

### Transcriptome analysis

All samples were integrated according to an established workflow described before (Angel et al. 2020). Briefly, for each experiment raw data files were downloaded from GEO, which were then subjected to background correction and probe-to-probeset (gene) summarization according to the robust multi-array (RMA) algorithm (Irizarry et al. 2003). Since samples were analyzed on multiple Affymetrix array platforms, only those genes were kept that were probed for on all array platforms. This resulted in the inclusion of 11,203 unique shared genes. Per array resulting non-normalized expression estimates of these 11,203 genes were then transformed into rank percentile values, in which the gene with the highest expression estimate was assigned the value of 1 and the lowest expression estimate was set to 0. All expression estimates in between were given a value based on the ranking of expression i.e., 0.01, 0.02, etc. with the steps in between adjusted to the number of genes, and genes with the same expression level were given the same value based on the average rank if they were not tied (i.e., tied for the value of 0.01 would give both a value of 0.015 in case each step was 0.01) (Angel et al. 2020). The dataset analyzed in this study consisted of 100 samples, and from each sample expression data of 11,203 genes was extracted. On this shared transcriptome, three different analyses were performed. (1) A multi-level principal component analysis, in which array type was used as blocking variable, performed using the Bioconductor package PCAtools (version 2.6.0) (Blighe and Lun 2022). Based on the Elbow method (Thorndike 1953), the relevant number of PCs was determined, (2) The top 10% most variable genes were visualized in a heatmap using the package *pheatmap* (v1.0.12), 3) To quantify and interpret sources of variation, the package variancePartition (version 1.26.0) was used (Hoffman and Schadt 2016). This package uses a linear mixed model (LMM) to quantify variation in gene expression attributable to biological or technical variables. To fit the normality assumption of an LMM, the rank percentile values were first transformed using the probit function (Angel et al. 2020). The genes of which the variance was explained for at least 40% by one of the variables were related to biologically meaningful changes using gene set overrepresentation analysis (ORA) applying a one-sided Fisher’s exact test (Draghici et al. 2003). Gene sets were retrieved from the expert-curated Kyoto Encyclopedia of Genes and Genomes (KEGG) database (Kanehisa et al. 2017) or Gene Ontology: Biological Processes (GOBP) (Ashburner et al. 2000; Gene Ontology 2021). ORA was performed using the package *clusterProfiler* (v4.3.3) (Wu et al. 2021).

## Results

### Study collection pipeline retrieves 27 unique studies, of which 13 used Affymetrix platforms

Our search strategy to identify studies that performed transcriptome analysis on Caco-2 cells retrieved 330 GEO Series (*i.e.,* unique GSEids) (Fig. 1 and Online Resource 1). Next, 176 series were excluded as in these studies regular wells were used as these Caco-2 models lack the presence of a basolateral compartment, which limits the investigation of transport and (anoxic) host–microbe interactions (Balimane and Chong 2005; Sambuy et al. 2005). Other reasons to exclude series were the following: transcriptomics was performed on or including other cell types, *e.g.,* immune cells and microbial cells not separated from Caco-2 (*n* = 64); no proper control condition was included (*n* = 5); data had been taken from a previously deposited dataset (*n* = 1) or data had not been published (yet) and study details could not be retrieved (*n* = 59). Of the latter category, the description of six series in NCBI pointed at the use of inserts or advanced in vitro models, of which two could be retrieved via other search strategies. We included two series of our own (NCBI-submitted) work (GSE158620 and GSE173729), resulting in a total number of unique series corresponding to 27 studies or papers. Across series, different array manufacturers and platforms were used. Five series had to be excluded because of a small number of unique genes analyzed by the platform used *(g* < 15,000) (Online Resource 1). Affymetrix platforms comprised the largest group, including 13 series across seven different platforms. Illumina and Agilent platforms were used in five series each (five and two different platforms, resp.) and one series was analyzed on the Stanford SHCU platform (“Others”). For our analysis, we decided to only continue with the series analyzed on Affymetrix platforms, because this group comprised a wider range of in vitro models with multiple conditions per model.

### Data extraction results in 100 samples, of which 75% were derived from inserts

**Fig. 2:**
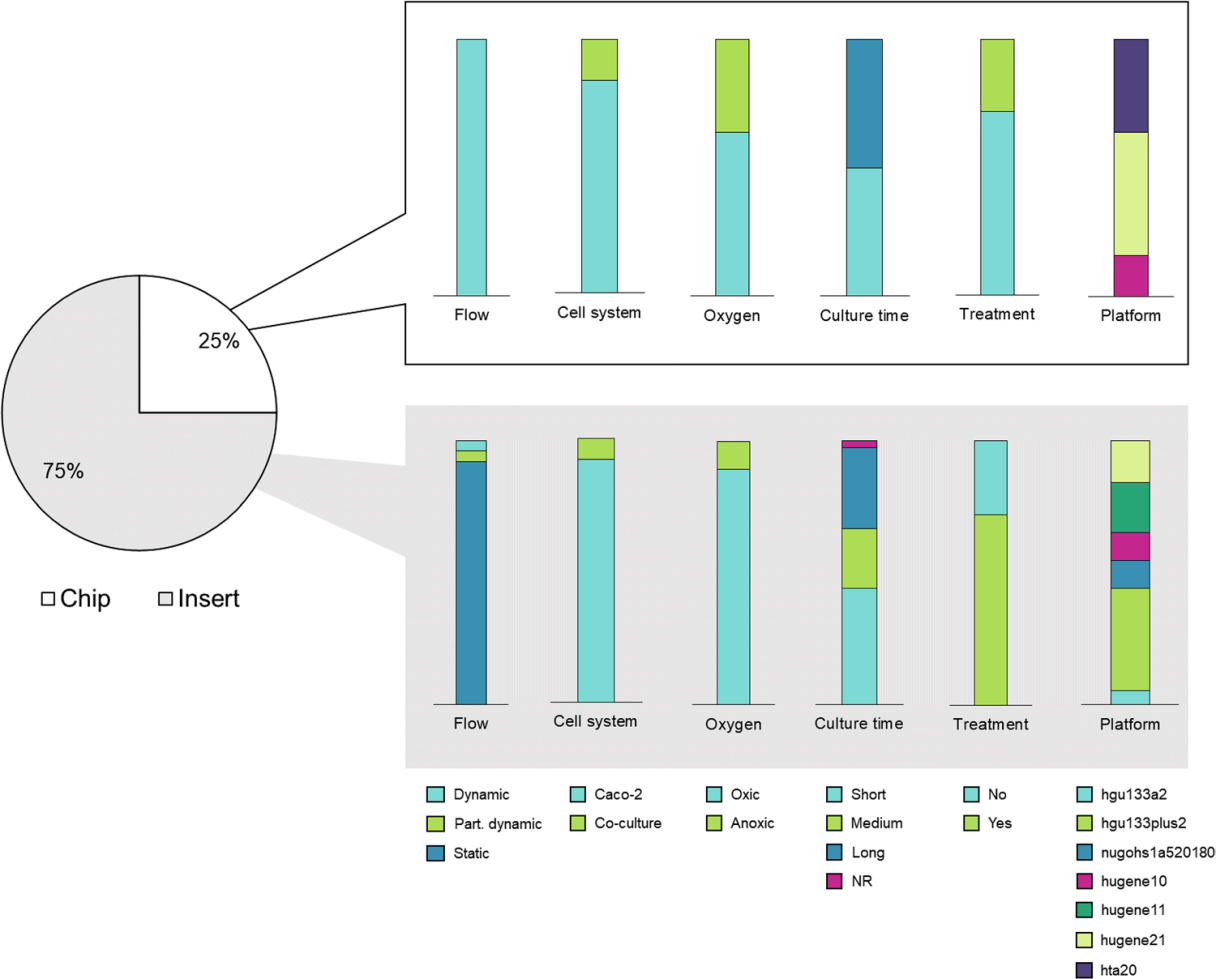
Overview of samples per model. Pie chart and bar chart represent no. of samples as percentage of total samples (*s* = 100) analyzed on an Affymetrix platform. For simplicity, the variable “Microbiome” is only divided into “Yes” and “No”. *NR* not reported, *hta20* Human Transcriptome Array 2.0, *hugene21* Human Gene 2.1 ST Array, *hugene11* Human Gene 1.1 ST Array, *hugene10* Human Gene 1.0 ST Array, *nugohs1a520180* NuGO array (human) NuGO_Hs1a520180, *hgu133plus2* Human Genome U133 Plus 2.0 Array, *hgu133a2* Human Genome U133A 2.0 Array

From the collected papers, we manually extracted the experimental set-up of each study (Online Resource 1). After selecting only samples encompassing Caco-2 cells grown under proper control conditions (*i.e.,* regular cell medium) or Caco-2 cells exposed to non-pathogenic intestinal bacteria or their bacterial products, we ended up with 100 different samples, including replicates (Online Resource 1). Of all 100 samples, 75% were derived from Caco-2 cells grown on inserts and 25% from Caco-2 grown on chips (Fig. 2). Note that the model used in GSE8187 (a 96-wells insert with flow) was classified as insert, as opposed to the semantics used by the authors (Sakharov et al. 2019). None of the studies that used an Affymetrix platform cultured Caco-2 as spheres. Across all 100 samples, different culture times were applied, ranging from 4 to 21 days (Online Resource 2 for full experimental set-up per study), which were further divided into short, medium, or long. One study on inserts did not report the time point of analysis. Samples were further categorized based on “GSEid”, “Microbiome”, “Oxygen”, “Flow”, “Cell system”, “Device” and “Platform” (Table 1).

**Table 1:** Overview of Affymetrix studies used for final transcriptome analysis. Studies are presented in chronological order. Each row represents a separate model

GSEid	Reference	Platform	No. of samples	Device	Cell system	Flow	Oxygen	Culture time	Microbiome? If yes, species and number
GSE7259	(Dihal et al. 2007)	Human Genome U133A 2.0 Array	4	Insert	Caco-2	Static	Oxic	Short; Medium	No
GSE15636	(Putaala et al. 2010)	Human GENOME U133 PLUS 2.0 ARRAY	17	Insert	Caco-2	Static	Oxic	Short	*L. acidophilus NCFM* (Bacterium 1, strain B and SN), *B. lactis* (Bacterium 4 and SN), *L. salivarius* (Bacterium 5 and SN)
GSE17625	(Ishimoto et al. 2010)	Human Genome U133 Plus 2.0 Array	6	Insert	Co-culture	Static	Oxic	Medium	No
GSE21976	(Turroni et al. 2010)	NuGO array (human) NuGO_Hs1a520180	8	Insert	Caco-2	Static	Anoxic apical compartment	Short	*B. bifidum* PRL2010 (Bacterium 7)
GSE30292	(Christensen et al. 2012)	Human Genome U133 Plus 2.0 Array	3	Insert	Caco-2	Static	Oxic	Long	No
GSE30364	(Rossi et al. 2013)	Human Genome U133 Plus 2.0 Array	3	Insert	Caco-2	Static	Oxic	Medium	No
GSE65790	(Kim et al. 2016)	Human Gene 1.0 ST Array	2	Insert	Caco-2	Static	Oxic	NR	No
		Human Gene 1.0 ST Array	4	Chip	Co-culture	Dynamic	Oxic	Short	VSL#3 (Bacterial community 1)
GSE79383	(Shah et al. 2016)	Human Transcriptome Array 2.0	9	Chip	Caco-2	Dynamic	Anoxic apical compartment	Short	*L. rhamnosus* GG (Bacterium 7), LGG + *B. caccae* (Bacterium 7 and 8), separated by PC membrane
GSE81867	(Sakharov et al. 2019)	Human Gene 1.0 ST Array	3	Insert	Caco-2	Static	Oxic	Short	No
		Human Gene 1.0 ST Array	3	Insert	Caco-2	Part. Dynamic	Oxic	Short	No
GSE115022	(Lépine et al. 2018)	Human Gene 1.1 ST Array	8	Insert	Caco-2	Static	Oxic	Long	*L. acidophilus* W37 (Bacterium 1, strain A), *L. brevis* W63 (2), *L. casei* W56 (3)
GSE156269	(Kulthong et al. 2021a)	Human Gene 2.1 ST array	4	Insert	Caco-2	Static	Oxic	Long	No
		Human Gene 2.1 ST array	4	Chip	Caco-2	Dynamic	Oxic	Long	No
GSE158620	(Kulthong et al. 2021b)	Human Gene 2.1 ST Array	8	Chip	Caco-2	Dynamic	Oxic	Long	No
		Human Gene 2.1 ST Array	8	Insert	Caco-2	Static	Oxic	Long	No
GSE173729	(Elzinga et al. 2021)	Human Gene 1.1 ST Array	3	Insert	Caco-2	Static	Oxic	Medium	No
		Human Gene 1.1 ST Array	3	Insert	Caco-2	Dynamic	Oxic	Medium	No

### Multi-level PCA reveals weak correlation of experimental variables with shared transcriptome

**Fig. 3:**
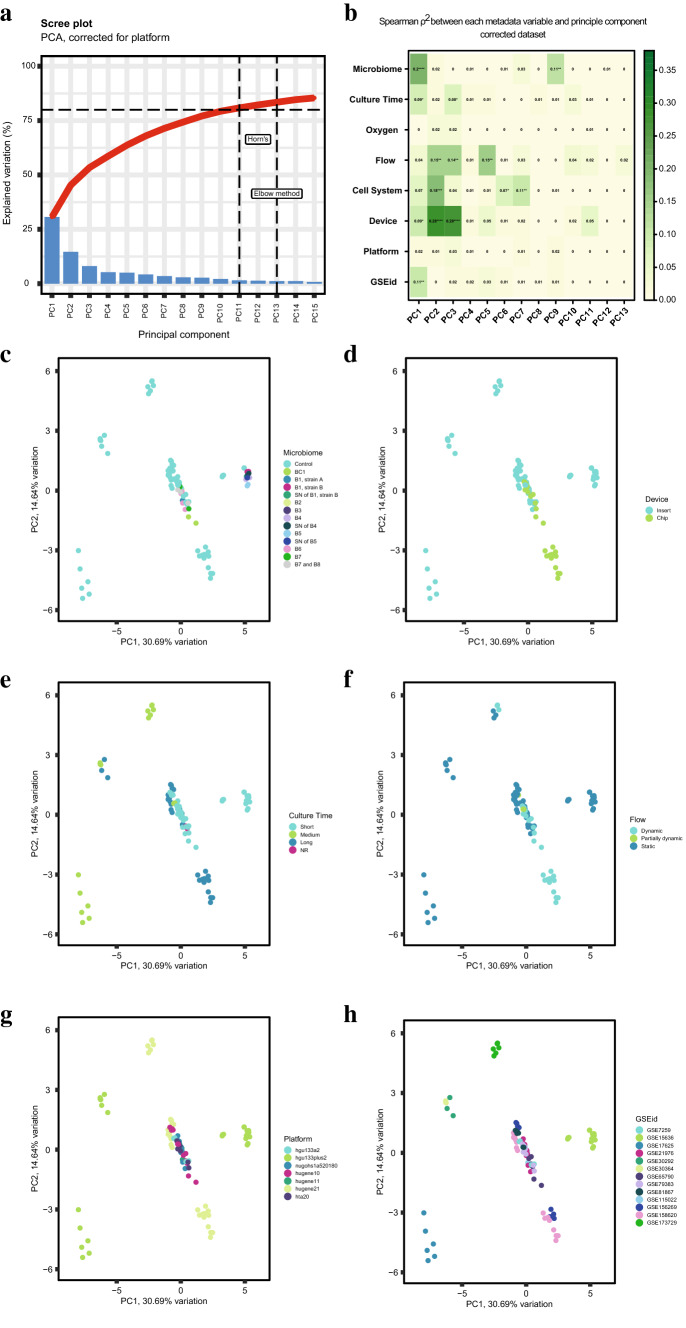
Multilevel principal component analysis at model level. After correction for platform, a principal component analysis was performed on a total of 11,203 shared genes, distinguishing eight experimental variables. **a** Scree Plot visualizing the variation explained by 18 PCs. **b** Spearman correlation *ρ*^2^ per model variable for the first 13 PCs. PCA plots of the first two components are provided and labeled by **c** microbiome (further defined in Table 1), **d** culture time, **e** device, **f** model, **g** array platform and **h** GSEid. PCA plots of oxygen and cell system can be found in Online Resource 3. *p < 0.05; **p < 0.01; ***p < 0.001; ****p < 0.0001. B# = Bacterium #; BC# = Bacterial Community #; *SN* supernatant, *NR* not reported; *hta*20 Human Transcriptome Array 2.0, *hugene*21 Human Gene 2.1 ST Array, *hugene*11 Human Gene 1.1 ST Array, *hugene*10 Human Gene 1.0 ST Array, nugohs1a520180 NuGO array (human) NuGO_Hs1a520180, *hgu133plus2* Human Genome U133 Plus 2.0 Array, *hgu133a2* Human Genome U133A 2.0 Array

First, we looked at the contribution of the eight pre-defined experimental variables to gene expression profiles, after controlling for the different Affymetrix array platforms. A multi-level PCA was performed (Fig. 3) on the maximum number of genes shared by all array platforms (*g* = 11,203 genes), referred to as the “shared transcriptome”. Based on the Elbow method (Thorndike 1953), we only considered the first 13 principal components (PCs) (Fig. 3a, b, Online Resource 3), of which PC1 accounted for 31% and PC2 explained 15% of the variation in the dataset. Overall, correlation coefficients were low, indicating weak to moderate correlation (Akoglu 2018). We report all variables separately below, starting with the highest correlations on PC1, 2 and 3.

“Microbiome” was the variable that contributed most significantly to the variation explained by PC1 (*ρ* = 0.20, *p* < 0.0001, Fig. 3b). Visualization of PC1 against PC2 demonstrated a separate cluster formed by three different bacterial species and their supernatant on PC1. These microbial exposures (See Table 1 for specifications) were all applied within one study (Putaala et al. 2010), but also clustered together with the control condition of that respective study (Fig. 3c, see GSE15636 in Fig. 3h). The variable “Device” contributed significantly to the variation explained by PC2 and PC3 (*ρ*^2^ = 0.28 and 0.28, resp., *p* < 0.0001, Fig. 3b). Visualization of PC1 against PC2 demonstrated a grouping of the chips, while the inserts were less congruent (Fig. 3d). Additionally, within the cluster of chips, a separation was observed between the short- and long-term cultured Caco-2 cells on chip (Fig. 3b**, e**), whereas in general, the variable “Culture Time” contributed significantly, but relatively weakly to the variation explained by PC1 and PC3 (*ρ*^2^ = 0.09 and *ρ*^2^ = 0.08, *p* < 0.05). Co-culture with other cell types contributed significantly to multiple PCs, mostly to PC2 (*ρ*^2^ = 0.18, *p* < 0.001) (Fig. 3b, Online Resource 3). The (partial) presence or absence of flow contributed significantly to the variation explained by multiple PCs, with a similar contribution to PC2, PC3 and PC5 (*ρ*^2^ = 0.15, 0.14 and 0.15 resp., *p* < 0.01). Visualization of the first two principal components, showed that the (partially) dynamic conditions clustered together, except for one study using Semi-Wet interface with Mechanical Stimulation (Fig. 3f, see GSE173729 in (Fig. 3h). Interestingly, none of the PCs were significantly associated with the variable “Oxygen” (Fig. 3b, Online Resource 3). Array platform did not contribute significantly to any principal component (Fig. 3b and g), demonstrating the successful correction of inter-platform differences (Online Resource 3 for uncorrected data). Finally, GSEid contributed significantly but weakly to the variation explained by PC1 (Fig. 3b and h).

**Fig. 4:**
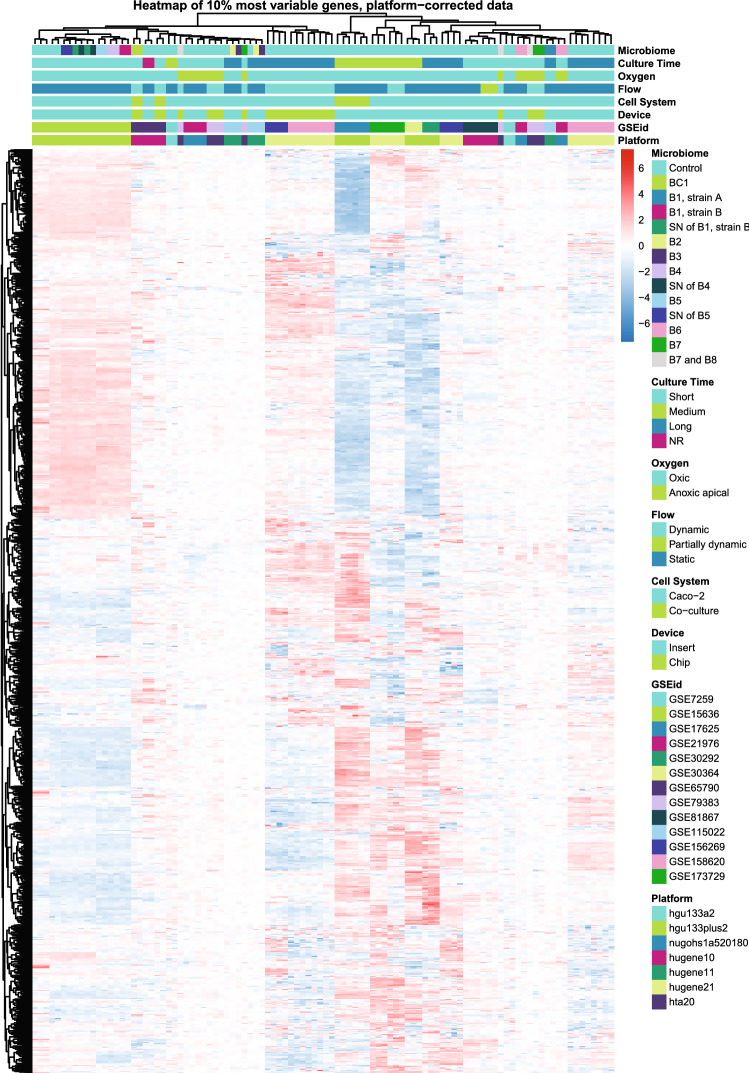
Clustering of samples (*s* = 100) based on the expression of the top 10% most variable genes shared between samples (*g* = 1122). Gene names are left out for readability. Heatmap represents relative gene expression varying from low (blue) to high (red). B# = Bacterium #; BC# = Bacterial Community #; SN = supernatant; hta20 = Human Transcriptome Array 2.0; hugene21 = Human Gene 2.1 ST Array; hugene11 = Human Gene 1.1 ST Array; hugene10 = Human Gene 1.0 ST Array; nugohs1a520180 = NuGO array (human) NuGO_Hs1a520180; hgu133plus2 = Human Genome U133 Plus 2.0 Array; hgu133a2 = Human Genome U133A 2.0 Array

Complementary to the multi-level PCA, a clustered heatmap was generated based on the 10% most variable genes of the shared transcriptome (Fig. 4). This analysis did not show clear groupings according to one of the variables. The clustered heatmap shows that the samples from two comparative studies between inserts and chips from Kulthong et al*.* (“GSEid”: GSE156269 and GSE158620) (Kulthong et al. 2021a, 2021b) clustered based on the device, in line with what was found by PCA. Moreover, the clustered heatmap confirmed separate clustering of the long- from the short-term cultured cells on chip.

### Variance partition analysis reveals high contribution of “GSEid” to individual gene expression

**Fig. 5:**
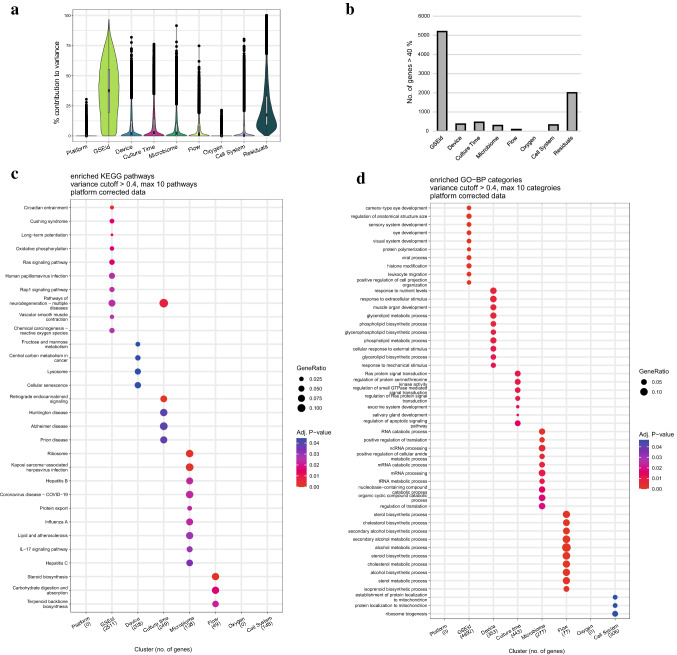
Variance partition analysis of all genes shared between samples (*s* = 100, *g* = 11,203). **a** Violin plot shown the percentage contribution of each variable to the expression of all genes. Based on the uncorrected data (Online Resource 4), a cut-off of 40% was chosen. **b** Number of genes of which contribution of respective variable was more than 40%. The overrepresentation of pathways within these genes in **c** KEGG Pathways and **d** GO-BP are displayed per variable

Next, we focused specifically on the genes of which variance of expression was explained by one of the eight specified experimental variables, by variance partition analysis (Hoffman and Schadt 2016). Without correction for “Platform”, the analysis revealed that the variance per gene was explained to the largest extent by “Platform” with an average of 43% across all genes (*g* = 11,203) (Online Resource 4). After correction for “Platform”, the variance of genes was explained mostly by “GSEid” (average of 38%), followed by residual, undefined parameters (25%) and “Device” (8%, Fig. 5a). Because of the relatively high contribution of “GSEid”, a technical parameter, we decided to focus only on genes of which variance was explained by one of the variables for more than 40%. The number of genes fulfilling this criterion varied from 0 (for “Oxygen”) to 5,198 (for “GSEid”) (Fig. 5b). The variance in the relatively high number of genes explained by residual parameters, can be explained by other model parameters that we extracted from the respective studies, but could not be included in the variance partition analyses, for instance used cell passages, membrane on which cells were seeded, membrane pore size, seeding area and seeding density. Overall, these model parameters were heterogeneous across and within in vitro models or were not reported at all by studies (Online Resource 4).

Next, we checked per variable which biological processes were enriched among the identified genes, *i.e.,* to which pathway(s) these genes were mapped more often than would be expected by chance. We used the KEGG and GOBP databases to retrieve the enriched pathways and biological processes, respectively. Only four variables resulted in significant overrepresentation of KEGG pathways, *i.e.,* "GSEid”, “Culture Time”, “Microbiome” and “Flow”. Overrepresentation analysis of genes of which variance was explained for more than 40% by “GSEid”, resulted in a heterogeneous mix of non-intestine-related KEGG pathways, which was the same for “Culture time” (Fig. 5c). Although the microbial exposures included in our dataset concerned non-pathogenic bacteria, overrepresentation analysis of the genes of which the variance was explained to a considerable extent by “Microbiome” revealed pathways related to host–pathogen interactions, such as “Kaposi sarcoma-associated herpesvirus infection”, “COVID-19”, “hepatitis B and C”, “Influenza A” and “IL-17 signaling pathway”. Genes of which the variance was explained mainly by “Flow” were enriched in the pathways involved in steroid and terpenoid backbone biosynthesis, but also in the breakdown and absorption of carbohydrates indicating an effect on energy homeostasis.

A similar approach was taken for Gene Ontology Biological Process (GO-BP) categories (Fig. 5d), which resulted in overrepresentation of categories for six variables (“GSEid”, “Device”, “Culture time”, “Microbiome”, “Flow” and “Cell system”). Biological processes overrepresented in "GSEid" included a wide variety of processes, with some clearly unrelated to the intestine as they pertain to the development of other organs. Among the rest, there was a focus on stress and adjustments in the cell via, for example, "histone modification" and “regulation of apoptotic signaling pathway” and two immune-related processes, “viral process” and “leukocyte migration”. Among "Device", "Microbiome", and "Flow", a range of different metabolic and biosynthetic processes showed up, many related to lipid metabolism. Process overrepresentation analysis for "Culture time" and “Cell System” resulted in several processes unrelated to the intestine or expected effects.

### Comparison of TEER values reveals heterogeneity in values and reporting quality

**Fig. 6:**
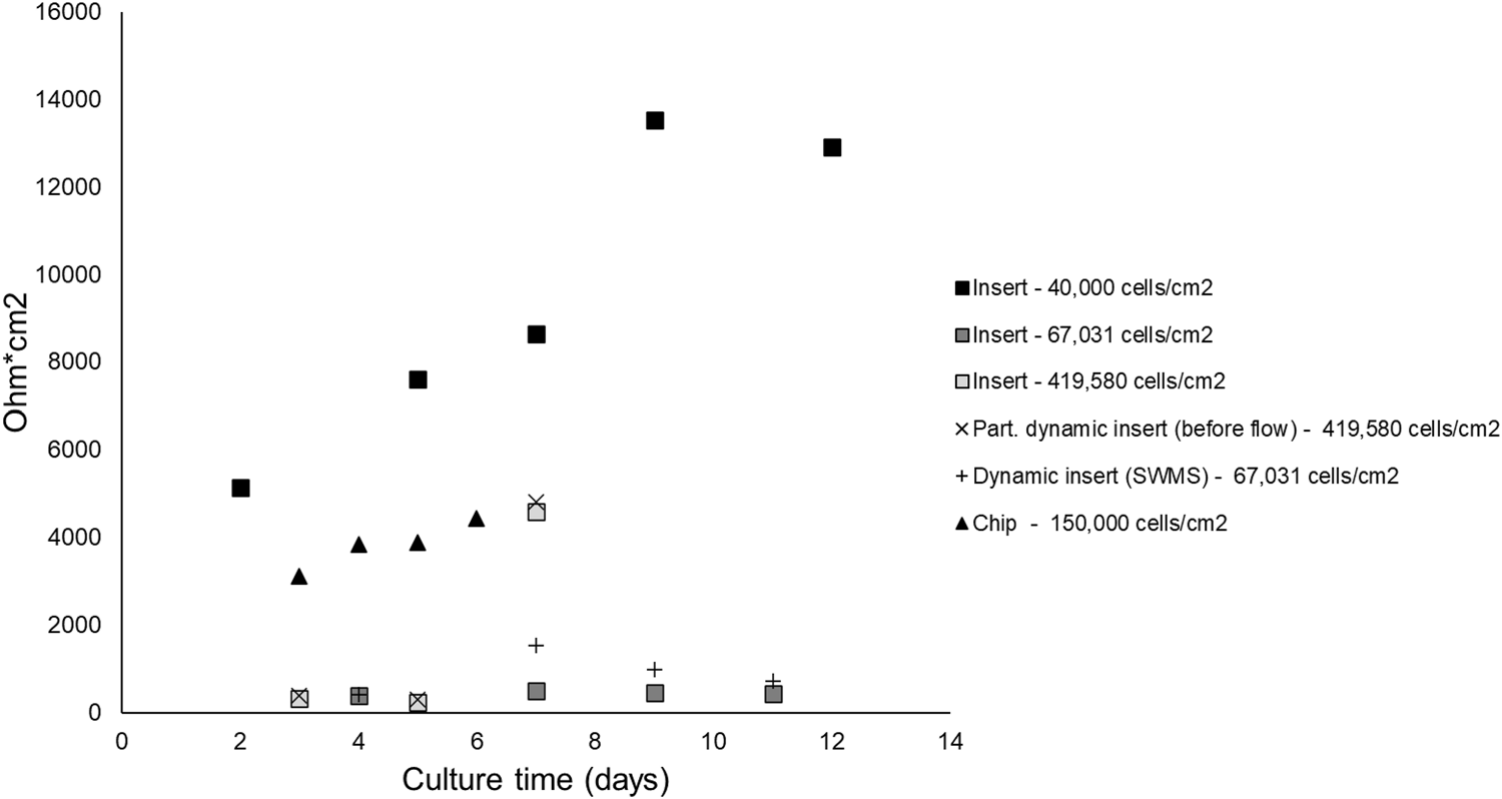
Trans-Epithelial Electrical Resistance across models. Only studies have been included which reported absolute TEER values (*n* = 4). Model conditions are presented individually, including initial seeding density. All conditions are oxic. Values were extracted from graphs using a digital ruler. Each data point represents the mean of 2–4 technical or biological replicates

Complementary to the transcriptome data, we collected additional functional experimental data from the identified studies to further characterize the used Caco-2 cell models. Among the identified studies, TEER was the only commonly reported outcome, which is a common measure of epithelial barrier integrity in in vitro studies using epithelial cell layers. TEER was reported in six Affymetrix studies comprising eight different models, of which one only reported the percentage of change in TEER, disabling comparison with other studies. Another study did not report seeding area, limiting calculations from Ohms/cm^2^ to Ohms * cm^2^. We extracted TEER values from six different models from four different studies across time points ranging from 2 to 12 days (Fig. 6), demonstrating a wide range of values.

## Discussion

In this study, we systematically compared transcriptomes of Caco-2 cells grown on permeable membranes using different in vitro systems modeling the human intestinal tract. We used the most frequently applied model, Caco-2 grown on inserts, as baseline for the comparison to other models incorporating an apical and basolateral compartment, allowing transport. Our shared transcriptome analysis indicated that of the studied parameters, the following had a significant, albeit relatively weak, influence on gene expression: the device in which Caco-2 cells were cultured; the presence of flow; and the exposure to non-pathogenic bacteria or their bacterial compounds. When looking at the individual gene level, however, variance in expression was mostly determined by the study (*i.e.,* GSEid). This points at a large heterogeneity in cell culture practices in in vitro models, which is supported by our analysis of experimental parameters from the respective studies (Online Resource 1), including variables, such as passage number, Caco-2 subclone and protocol used, all of which can influence the results (Briske-Anderson et al. 1997; Larregieu and Benet 2013; Sambuy et al. 2005; Zucco et al. 2005). Because these variables cannot be expressed as a number or concern an ambiguous range without a common starting point (*e.g.,* for cell culture passage), these could not be included in the analysis. Additionally, we demonstrated a lack of proper reporting of experimental variables, which has been stressed previously (OECD 2018). Our systematic analysis sets the scene for similar future analyses of (-omics) data between in vitro models, and therefore is a next step toward transparent reporting of in vitro studies to achieve an increased acceptance of non-laboratory animal study-derived data in life sciences.

Our multi-level PCA demonstrated that the variables “Flow” and “Device” had a similar effect. This is probably because dynamic samples were often derived from chips, except for three samples derived from inserts*.* These three concerned Caco-2 cells grown under Semi-Wet interface with Mechanical Stimulation, in which the cells are cultured under minimal liquid in the apical compartment and put on an orbital shaker (Navabi et al. 2013). Interestingly, visualization of PC1 against PC2 revealed that these samples clustered separately from the other dynamic samples (chips), but the same was true for the static control (insert) of this study (Elzinga et al. 2021), indicating that other factors explain the separate clustering of these samples. Overall, differences in (micro)fluidic design of included dynamic samples complicated inter-model comparison (hDMT 2021; Ma et al. 2021). Although most of the samples still clustered together, it would be informative to quantify shear stress or the resulting shear stress on the cells, as opposed to distinguishing only between static, dynamic and partially dynamic. Shear stress values were, however, not reported for all studies (except three Kim et al. 2016; Kulthong et al. 2021a, 2021b) or could, in some cases, not be calculated with the available information.

“Microbiome” was relatively strongly associated with (shared) gene expression, despite the heterogeneity in bacterial treatments tested in the included studies. For microbiome-specific effects of the tested bacteria, we refer to the individual papers corresponding to the studies (Species and corresponding references in Table 1). To facilitate co-culture of Caco-2 with intestinal (anaerobic) bacteria an in vitro model that is (at least) partially anoxic is required. Remarkably, our analyses demonstrated that the partial lack of oxygen did not seem to influence Caco-2 gene expression in the included in vitro models at all. For the studies that included microbes, the actual concentration of oxygen in the apical compartment was not always quantified. Therefore, the question remains whether the lack of association of oxygen with gene expression is due to failure of the respective studies to create an anoxic atmosphere in the apical compartment or that the absence of oxygen on one side simply does not affect gene expression of Caco-2 cells (*e.g.,* because oxygen supply via the other side is sufficient). It would be advantageous for future studies to monitor and report O_2_ concentrations, as has been done already in inserts (Ulluwishewa et al. 2015) as well as more advanced in vitro models (Jalili-Firoozinezhad et al. 2019).

Because we observed a large heterogeneity in culture time between studies, we decided to define three groups based on cell culture time ranges. The definition of total culture time varied between studies. For instance, Dihal et al*.* accounted for the pre-confluent phase of cells once seeded, by starting to count from day 2 after seeding (Dihal et al. 2007), whereas other studies considered the time of seeding as the starting point. In the case of “Culture time” there is a consensus in literature that there is a direct relation with differentiation and thus, gene expression. Although the exact time until a plateau is reached differs for each differentiation marker, the generally accepted culture time for full differentiation is 21 days (Hubatsch et al. 2007; Natoli et al. 2012; Sambuy et al. 2005; Vachon and Beaulieu 1992). In our dataset, total culture times differed between studies using inserts, which could depend on the study aim. For instance, for studying barrier properties, fifteen days of culture was shown to be sufficient (Zucco et al. 2005). However, we observed no strong influence of the variable “Culture time” on gene expression in our PCA. This could be due to the inclusion of chips and studies with adjusted protocols, both reported to change the relationship between culture time and differentiation (Kim and Ingber 2013; Kim et al. 2014; Natoli et al. 2011). Among others, the shear stress that cells experience on chips has been described to enhance differentiation of intestinal cell lines, thereby reducing the total culturing time (Kim and Ingber 2013; Kim et al. 2014). This became apparent within the group of chips, where the short-cultured cells were separated from the long-term cultured cells in both the PCA (PC1 against PC2) and the heatmap cluster analysis. Within inserts, the lack of association between “Culture time” and gene expression is likely due to the inclusion of studies such as GSE30292, in which cells were maintained at low density by subpassaging cells at 50% confluence, instead of the density prescribed by ATCC (between 80 and 90%) (ATCC 2021; Christensen et al. 2012) Subsequently, these cells were cultured for a long period on inserts, *e.g.,* three weeks showing a profound effect on gene expression over time. Interestingly, both our PCA and heatmap cluster analysis demonstrated that these samples clustered together with medium-term cultured cells, and not with other long-term cultured cells (all maintained at normal density before seeding). It was already shown previously that low-density cells, although expressing the same level of differentiation markers as high-density (90%) grown cells, have a slower exit from cell cycle, including a delay in downregulation of cyclin A and increase of differentiation marker sucrase (Natoli et al. 2011). Although low-density grown cells should have differentiated to the same extent as high-density grown cells after three weeks (ATCC 2021; Natoli et al. 2011), our data suggest that low-density maintained cells grown for a long period on inserts demonstrate a transcriptome profile more similar to high-density maintained cells grown for a medium period on inserts. This reinforces the need for clear reporting on cell maintenance practice. Overall, the effects of, a.o., flow and seeding density on the interaction between culture time and gene expression, have potentially obscured the expected effect of culture time on gene expression. For future analyses, these interactions could be included as a separate variable in the PCA. This, however, requires proper quantification of these interactions, which is now hampered by model heterogeneity and incomplete reporting.

Our variance partition analysis provided an overview of which pathways or biological processes were associated with each of the selected variables. In the case of the KEGG pathways only four variables contributed to pathways. The pathways associated with "Microbiome" were immune system-related, though the microorganisms included in our study are non-pathogenic bacteria, while the pathways are associated with infectious diseases. This is likely because both groups of microbiota act on Toll-like and other pattern recognition receptors (Isolauri 2001; Perdigon et al. 1995), but with probably opposing downstream effects. We did not find similar processes associated in the GOBP analysis, where “Microbiome” was mostly associated with RNA and translation processes, which are rather broad. The parameter “Flow” was only associated with two pathways, *i.e.,* steroid and terpenoid backbone synthesis, and these were supported by the GOBP analysis, where “Flow” associated with many processes related to these pathways. Similar GOBPs were enriched in the genes mainly determined by “Device”, which is likely due to the overlap between these two variables, as discussed previously. Both the variables “Culture time” and “Cell System” were associated with several RNA-related processes but did not show a clear direction of effect in the other processes. Better harmonization of in vitro models or more accurate categorization of models (including more variables, see below), would probably point at more specific categories of pathways and processes, with a higher overlap between the KEGG and GOBP analysis.

The relatively low correlation coefficients in the PCA, as well as the high number of genes of which variance was explained to a substantial extent by residual variables, indicated that a substantial number of variables is still missing in our analysis. Future analyses would be stronger by including other experimental variables like passage number, seeding density, and seeding membrane. Our systematic extraction of these parameters revealed a large heterogeneity between studies, making it impossible to reach significant outcomes when these parameters are used as inputs. We extracted other parameters as well, such as cell medium composition in terms of fetal calf serum, antibiotics, and other supplements—specific clones used and medium refreshment frequency, demonstrating similar heterogeneity. All these parameters have been reported to affect Caco-2 cell behavior (Sambuy et al. 2005), and therefore should be reported when publishing data, as also suggested by the MIAME and MINSEQE guidelines (outlining the Minimum Information About a Microarray Experiment or Minimum Information About a Next-generation Sequencing Experiment that should be included when describing a microarray or sequencing study) (Brazma et al. 2012, 2001). In general, the degree to which data were deposited in MIAME- or MINSEQE-compliant public data repositories, like NCBI Geo and ArrayExpress, limited the availability of our transcriptome-centered approach. For instance, we encountered studies that had not deposited their data in these repositories (Greenhalgh et al. 2019; Kim et al. 2014) and vice versa, data series which had not been linked to the correct study (see number of retrieved studies Fig. 1). We cannot determine how many relevant, unpublished studies we missed, since the description in NCBI on the exact in vitro model used was not always complete. In the context of the increasing global interest in Open Science, the importance of depositing open data in public repositories was recently stressed (Forero et al. 2021). Specifically for microarray gene expression analyses, researchers demonstrated limited repeatability of published microarray studies, which was due to inadequate reporting on the used methods and other factors like software unavailability, or unclear reporting of the results (Ioannidis et al. 2009). The quality and ability to reuse data from other end points has been complicated by reporting issues as well. This is exemplified by TEER measurements, the most reported outcome in our dataset (other than gene expression). We concluded, however, that even the reporting quality of TEER was low, *i.e.,* in terms of number of replicates used; culture area; normalization to blank inserts and temperature at which the measurement was performed. Note that in a few studies (Brazma et al. 2001), TEER was solely monitored as a quality measure of the monolayer, although the reported required minimum varied between studies (150–330 Ω cm^2^) or was not defined. Similarly, a standardized method to measure TEER on chip devices has not been established yet (Lépine et al. 2018; Rossi et al. 2013). Our data reinforce the need for standardized TEER protocols and reporting guidelines for inserts, chips and other devices that are currently being developed.

All variables taken together, the data used for this study reiterate the need for a universally accepted Caco-2 cell culturing method, which also includes proper reporting of all variables and read-outs. The lack of adequate reporting is a commonly known problem in in vitro research (Hartung et al. 2019), which limits the reproducibility and translatability of animal-free methods. Moreover, our study demonstrates that poor reporting quality of (meta)data also limits integration of existing in vitro data in systematic analyses across models or studies, re-emphasizing the need for Findable, Accessible, Interoperable, Reusable (FAIR) data guiding principles (Wilkinson et al. 2016). Additionally, the need to apply novel-approach methods in, for instance, chemical risk assessment as well as in fundamental and clinical research is increasing. In this context, the development of an in vitro critical appraisal (IV-CAT) tool to improve the peer review as well as the quality of published in vitro research, as proposed by De Vries and Whaley (de Vries and Whaley 2018) is highly appreciated.

In summary, our study aimed to compare transcriptome responses of Caco-2 cells in different in vitro models as systematically as possible. Our analysis comprised both a transcriptome-wide and gene-specific approach, has the potential to find associations of pre-defined experimental variables with gene expression and uncover biological pathways associated with these variables. We complemented this analysis with manual extraction of other data, including model parameters and functional outcomes such as TEER. In this way, this paper can serve as an example for future comparison to in vitro models. Currently, controls are designed with only their own experiment in mind, failing to consider variables that are important to allow for comparison to other studies. More importantly, the results show the need for standardization and benchmarking of both current and future in vitro models. This should start with proper reporting of model parameters (Emmerich and Harris 2020), as only in this way research can be reproduced and compared. We acknowledge that benchmarking of an in vitro model depends largely on the research question, *e.g.,* whether the in vitro model is used for risk assessment, drug development or uncovering fundamental biological knowledge. Current approaches might still function in cases where you compare a potent exposure to controls across studies, but it fails to allow for extraction of more subtle effects of (underreported) model and experimental variables, reducing potential value of data. For current in vitro methods, the OECD is already working toward improving models via GIVIMP, setting standards on models and reporting (OECD 2018). At the same time, there the is also a push to apply (part of) this knowledge in organ-on-chip technology in (to-be-)developed models (hDMT 2021; Vollertsen et al. 2021) by standardizing chip design. But application in the development of these models should go further than just technical design. A continued push in this direction is key and responsibility lies with not only the researchers that should execute experiments according to existing guidelines (Hartung et al. 2019), but also the funding agents deciding to invest in the application or development of in vitro models as well as journal editors and peer reviewers critically evaluating the work. Only in this way, researchers will be able to unlock the full potential of in vitro models, to eventually reduce, refine, and replace the need for animal testing.

## Supplementary Information

Below is the link to the electronic supplementary material.

Supplementary file1 Summary of data regarding study collection and data extraction, including a) Retrieved 330 GEO Series (n = 330) in the GEO Database, including excluding criteria b) number of genes analyzed per array platform, including excluded platforms c) data extraction from all studies using Affymetrix platforms, including model specifications and experimental set-up d) sample overview of all samples (n = 100) analyzed on Affymetrix platforms (XLSX 79 KB)

Supplementary file2 Online Resource 2. Overview of experimental set-up per model analyzed on Affymetrix platforms. Multilevel principal component analysis at model level. After correction for platform, a principal component analysis was performed on a total of 11,203 shared genes, distinguishing eight experimental variables. Online Resource 3. a) Spearman correlation PC per model variable for the first 13 PCs. PCA plots of the first two components are provided and labeled by b) oxygen c) cells system. d) Spearman correlation ρ2 per model variable for the first 13 PCs without correction for platform. * p <0.05; ** p <0.01; *** p <0.001; **** p <0.0001. Variance partition analysis of all genes shared between samples (s = 100, g = 11,203). Online Resource 4. a) Violin plot shown the percentage contribution of each variable to the expression of all genes without correction for platform. Additional data was extracted per model, including b) membrane on which the cells were seeded, c) pore size of the membrane, d) membrane coating, e) seeding density, f) seeding area and g) passage number used. (PDF 959 KB)

## Data Availability

The datasets analysed during the current study have been generated by others and are available in the NCBI Gene Expression Omnibus (GEO), https://www.ncbi.nlm.nih.gov/geo/. The code to analyse the data is deposited online on Zenodo.org, https://zenodo.org/record/7525836#.Y77gohWZO3A.
