# Supplementary figures and images for: Systematic comparison of transcriptomes of Caco-2 cells cultured under different cellular and physiological conditions
Source: Arch Toxicol. 2023 Jan 21;97(3):737–53. doi: 10.1007/s00204-022-03430-y (PMC9862247; doi:10.1007/s00204-022-03430-y)

Online Resource 2

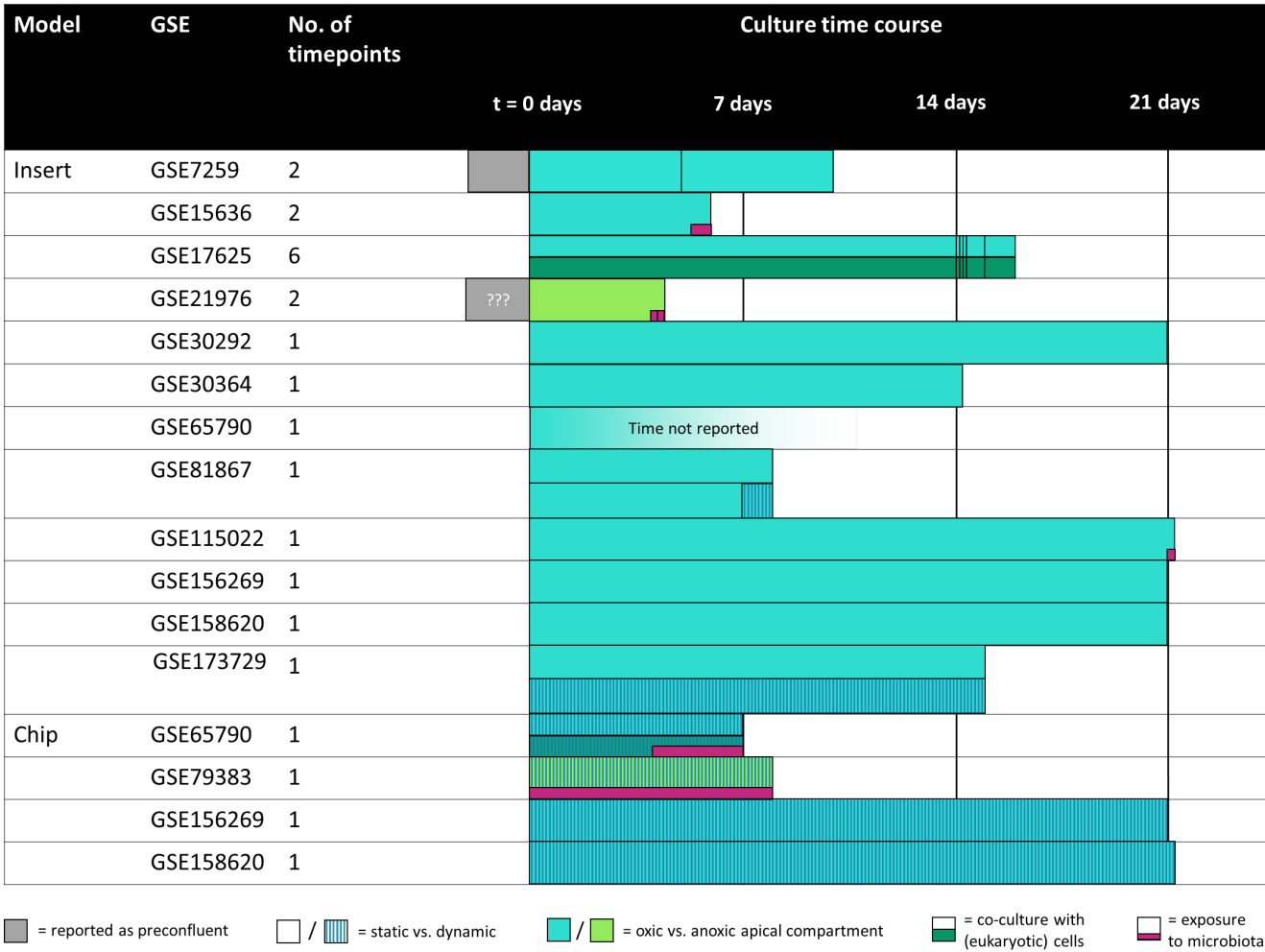

Online Resource 3

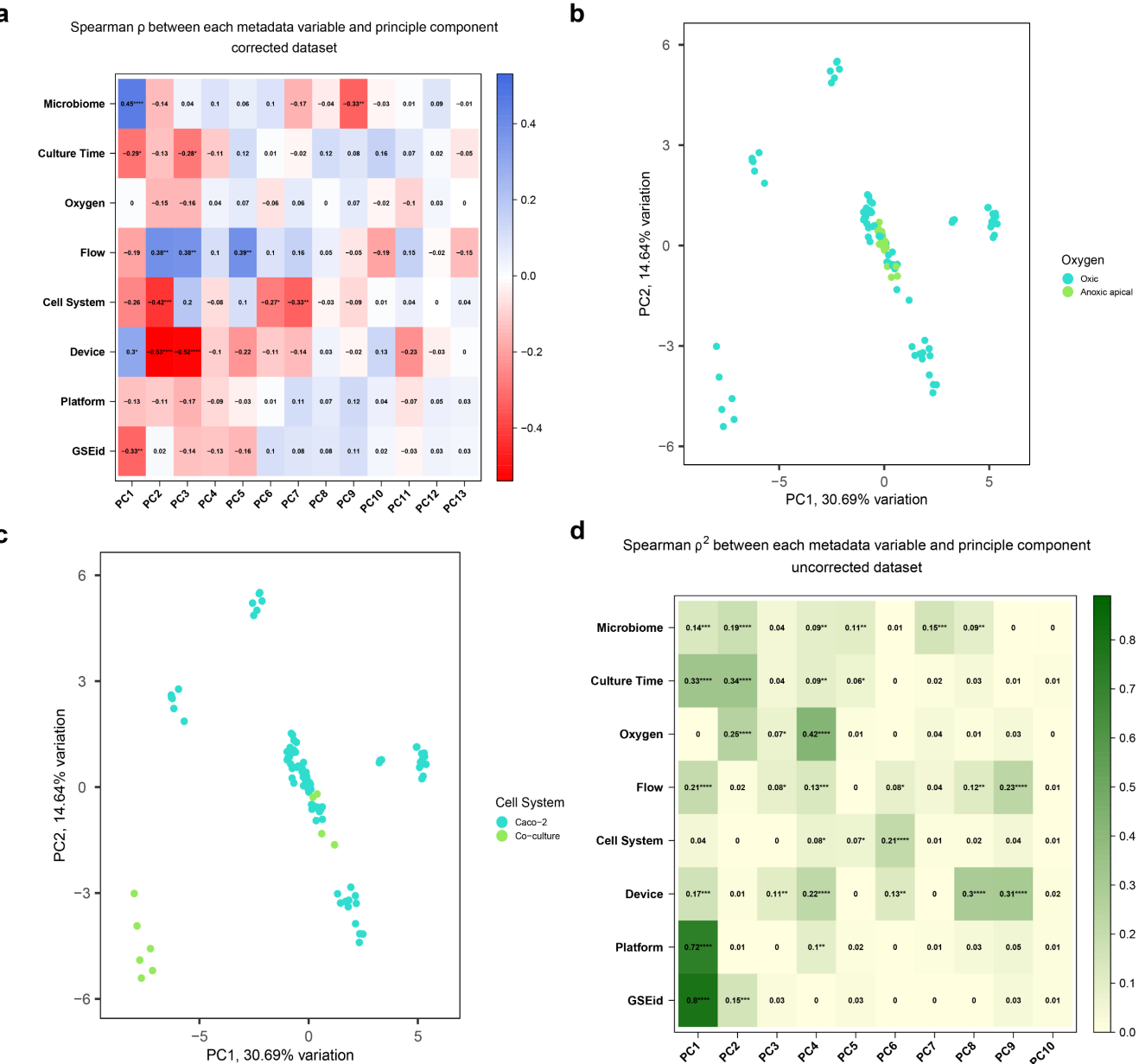

Online Resource 4

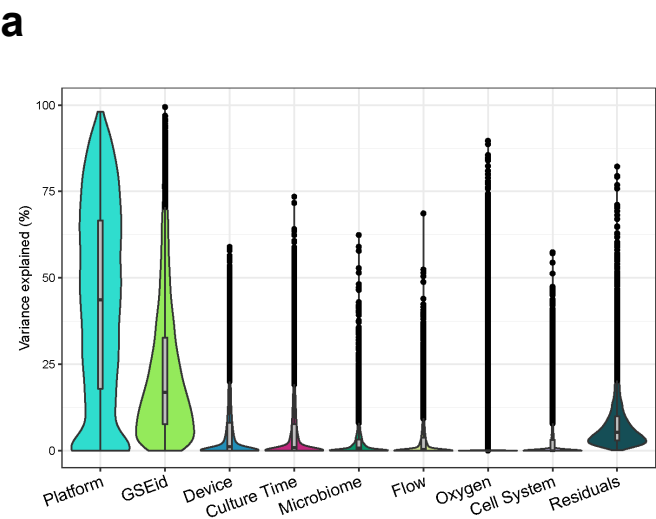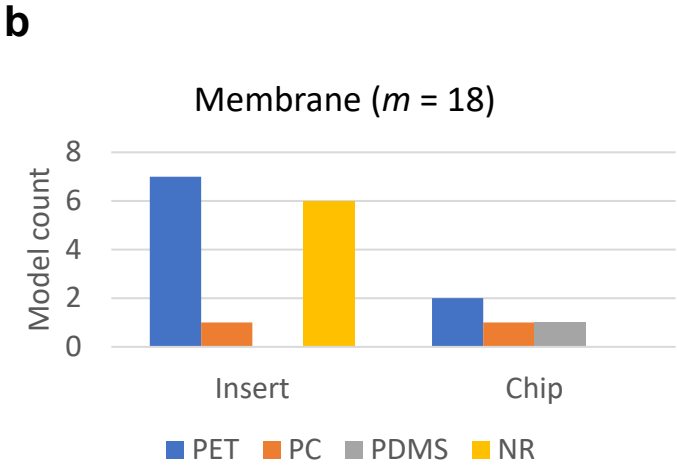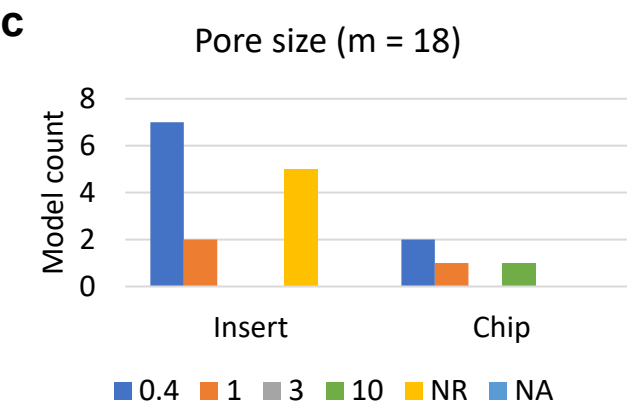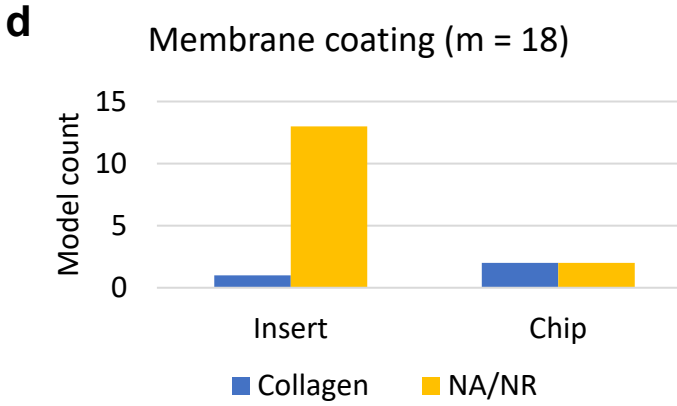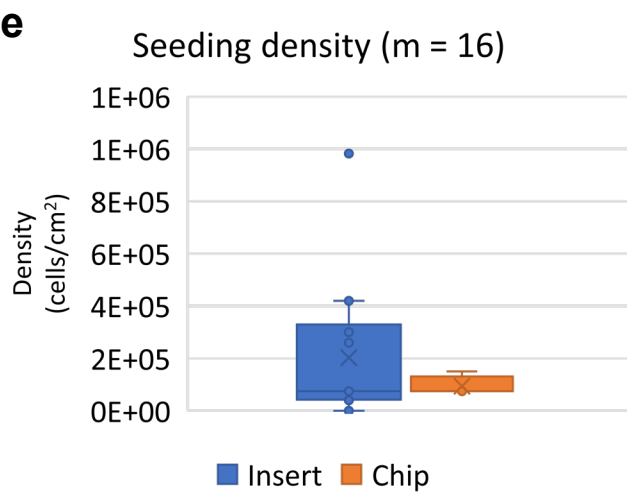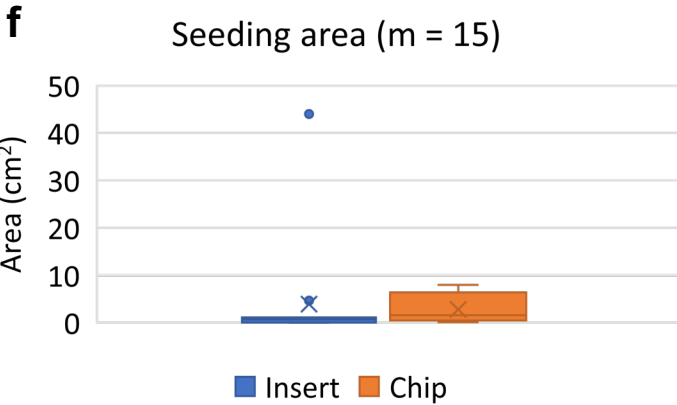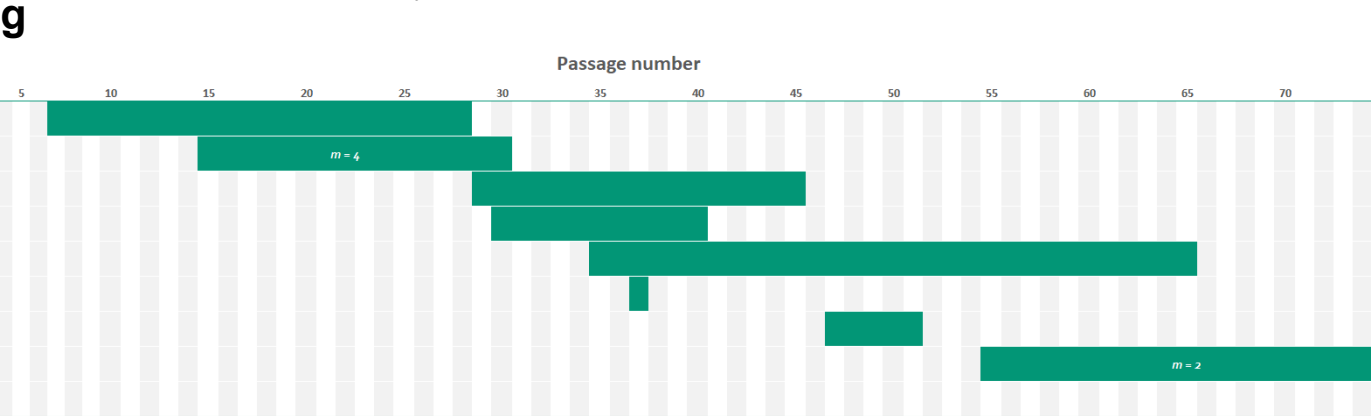

Supplement: Supplementary file 2 — Supplementary file2 Online Resource 2. Overview of experimental set-up per model analyzed on Affymetrix platforms. Multilevel principal component analysis at model level. After correction for platform, a principal component analysis was performed on a total of 11,203 shared genes, distinguishing eight experimental variables. Online Resource 3. a) Spearman correlation PC per model variable for the first 13 PCs. PCA plots of the first two components are provided and labeled by b) oxygen c) cells system. d) Spearman correlation ρ2 per model variable for the first 13 PCs without correction for platform. * p <0.05; ** p <0.01; *** p <0.001; **** p <0.0001. Variance partition analysis of all genes shared between samples (s = 100, g = 11,203). Online Resource 4. a) Violin plot shown the percentage contribution of each variable to the expression of all genes without correction for platform. Additional data was extracted per model, including b) membrane on which the cells were seeded, c) pore size of the membrane, d) membrane coating, e) seeding density, f) seeding area and g) passage number used. (PDF 959 KB) [file 204_2022_3430_MOESM2_ESM.pdf]
